# Predictors of Mother and Infant Emergency Department Attendance and Admission: A Prospective Observational Study

**DOI:** 10.1007/s10995-022-03581-5

**Published:** 2023-01-26

**Authors:** Amanda J. Mason-Jones, Luis Beltrán, Ada Keding, Vashti Berry, Sarah L. Blower, Karen Whittaker, Tracey Bywater

**Affiliations:** 1 Department of Health Sciences, University of York, Heslington, York, YO10 5DD UK; 2 College of Medicine and Health, South Cloisters, University of Exeter, St Luke’s Campus, Heavitree Road, Exeter, EX1 2LU UK; 3 School of Nursing, University of Central Lancashire, Preston, PR1 2HE UK

**Keywords:** Emergency, Reproductive health, Ethnicity, Respiratory disease, Inequity, Health care utilisation

## Abstract

**Objective:**

To explore the predictors of emergency department attendance and admission for mothers and their infants.

**Methods:**

Self-reported emergency department (ED) attendance and admission, sociodemographic, mental health, and other measures were recorded at baseline and at 12 months at 4 sites in England between May 2017 and March 2020.

**Results:**

Infants’ gestational age (OR 0.73, 95% CI 0.61 to 0.88, p = 0.001), mothers’ mental health (OR 2.40, 95% CI 1.30 to 4.41, p = 0.005) and mothers’ attendance at ED (OR 2.34, 95% CI 1.13 to 4.84, p = 0.022) predicted infant ED attendance. Frequency of attendance was predicted by ED site (IRR 0.46, 95% CI 0.29 to 0.73, p = 0.001) and mothers’ age (IRR 0.96, 95% CI 0.92 to 1.00, p = 0.028). Infant hospital admissions were predominantly for respiratory (40%) and other infectious diseases (21%) and were predicted by previous health problems (OR 3.25, 95% CI 1.76 to 6.01, p < 0.001).

Mothers’ ED attendance was predicted by mixed or multiple ethnic origin (OR 9.62, 95% CI 2.19 to 42.27, p = 0.003), having a male infant (OR 2.08, 95% CI 1.03 to 4.20, p = 0.042), and previous hospitalisation (OR 4.15, 95% CI 1.81 to 9.56, p = 0.001). Hospital admission was largely for reproductive health issues (61%) with frequency predicted by having attended the ED at least once (IRR 3.39, 95% CI 1.66 to 6.93, p = 0.001), and being anxious or depressed (IRR 3.10, 95% CI 1.14 to 8.45, p = 0.027).

**Conclusions for Practice:**

Improving the reproductive and mental health of mothers may help to avoid poor maternal and infant health outcomes and reduce emergency service utilisation and hospitalisation.

## Significance Statement

*What is already known on this subject?* The association between parental mental health and children’s health care utilisation has previously been reported and poverty may increase utilisation of emergency department services.

*What does this study add?* This study is the first known from the UK to explore predictors of mother and infant emergency department utilisation at the infant, mother, and health service level. Reproductive health issues may be an overlooked cause of emergency department attendance and admission for women and their infants. Inaccessible primary care services may lead to more use of emergency services.

## Introduction

The social determinants of maternal health have a direct physiological, psychological and developmental impact (Vameghi et al., 2018) on children’s health in the early years (Minkovitz et al., 2005) and on utilisation of emergency department (ED) services (Credé et al., 2020; Flynn et al., 2004; Morrison et al., 2013). Health emergencies for women can be related to acute events but can also linked to chronic, longer term issues for women that may become acute during pregnancy (Cunningham et al., 2017).

Children, especially those under a year old, who live with an adult with poor mental health show an increase in recurrent health problems themselves (Alvarez et al., 2015), are more susceptible to infections and injuries, and have higher rates of attendance at emergency departments (Dreyer et al., 2018; Paranjothy et al., 2018). However, the temporality of this association is not always possible to deduce from retrospective studies (Farr et al., 2013). Despite there being studies exploring links between maternal and infant health and emergency department utilisation from North America (Anderson et al., 2008; Farr et al., 2013), there are no similar studies from England or the UK that have explored the relationship between maternal physical, mental health and infant ED utilisation.

In 2014, the costs related to use of EDs and minor injury units in England was estimated to be £2.7 billion. Unplanned emergency admissions of children have been estimated to cost around £245 million per year to the NHS with the greatest costs being for those services providing care in the most deprived areas (Kossarova et al., 2017). The recent reported decline in ED attendance of very young children during the COVID-19 pandemic is potentially concerning (NHS England, 2020). Recent evidence from one Paediatric ED in England suggests that those children who did attend between March and April 2020, were younger and more severely ill compared to previous years (Rose et al., 2020).

Apart from the costs to the NHS (Kossarova et al., 2017), the burden of emergency health care for families includes time taken off work, waiting, travel, and childcare costs and increased stress and anxiety. Therefore, an understanding of the issues at multiples levels of the system is urgently required to explore what, if any, preventative measures might be developed.

The purpose of this study was to explore the predictors of emergency department utilisation, that is, attendance and associated admission (via ED) to hospital of mothers and their infants enrolled in the E-SEE study.

## Methods

This study was a planned and approved observational sub-study of the E-SEE Steps randomised controlled trial (Bywater et al., 2022a) which included a trial (Bywater et al., 2022b), process evaluation (Berry et al., 2022) and economic evaluation (Cox et al., 2022) where further information and detail about the measures, methods and findings can be found. The STROBE guidelines for reporting observational studies have been followed.

### Ethical Approval

The study was approved by NHS Wales Research ethics Committee (REC Reference 15/WA/0178, IRAS 173946) and the University of York (Reference number FC15/03). Participants gave written informed consent before taking part.

### Data Sources

Mother-infant dyads were recruited in May 2017 via self- or health practitioner referrals when the infant was 8 weeks of age or younger. Exclusion criteria included if the child had developmental difficulties. Participants were recruited in four English local authorities, two in the north of England, one in the Midlands, and one in the south of England. Data included for this study were collected at baseline and at two further timepoints when the infants were approximately 4 and 11 months of age. Final follow up data collection for E-SEE was completed in March 2020.

Baseline and follow up data from the following measures were included in this study: self-reported adverse events, hospital attendance and hospital admissions from the Client Service Receipt Inventory (CSRI), (Beecham & Knapp, 2001), quality of (caregiver) relationships, EuroQol quality of life inventory (EQ5D-5L) (Essink-Bot et al., 1997) and Parent Sense of Competence (PSoC) scale (Johnston & Mash, 1989).

### Patient and Public Involvement

A representative cross-site Parent Advisory Committee (PAC) was established to provide a parental perspective throughout the study. PAC members were consulted on various elements of study design. Their inputs contributed to the selection of measures and data collection procedures. Initial findings and final results were shared with a parents’ group and with study participants.

### Outcomes

The following self-reported data from the CSRI were used as study outcomes:

- Child hospital admission
- Mother hospital admission
- Child ED attendance
- Mother ED attendance

All outcomes were recorded as binary incidence (yes/no) and continuous frequency (number of separate occurrences) per mother–child dyad. Any duplicate records were identified by date and event details and were consolidated into unique events. Study data contained reasons for hospital admission (which were reported thematically using the first reported reason if there were more than one). No reason for ED department use were available.

The following predictors were included in the initial analyses based on their theoretical influence on emergency department use and admission from the literature. Based on the distribution of categorical variables in the data, some categories were combined (indicated below by [square] brackets) to allow viable and meaningful analyses.

- *Demographics (mother)*
  - Age (in years)
  - Ethnicity (white British, Asian/Asian British, Black/Black British, Mixed/Multiple, Other)
  - Employment status (paid employed [employed, maternity leave], unemployed [unemployed, homeworker], other [student, retired, other])
  - Number of biological children (including current child)
  - Age when the first child was born (in years)
- *Relationship information (mother)*
  - History of emotional or physical abuse by partner or someone else important (Yes, No), recorded at baseline and any incidences over all collected time points (Yes, No)
- *Self-reported outcome measures (mother)*
  - EQ5D-5L anxiety/depression (Anxious or depressed [slightly/moderately/severely/extremely anxious or depressed], Not anxious or depressed)
  - PHQ-9 Depression severity (score range 0 to 27, higher score indicating greater depressive symptoms)
- *Pregnancy and parenting information*
  - Being a first-time parent (Yes/No)
  - Health problems during pregnancy (Yes/No)
  - Problems or difficulties at the time of child's birth (Yes/No)
- *Infant characteristics*
  - Sex (male, female)
  - Gestational age (in weeks)
  - Premature birth (Yes/No)
  - Health problems or serious injuries since birth (Yes/No)

### Statistical Analyses

Baseline characteristics were summarised using descriptive statistics for the total study sample and mothers and children for whom one or more study outcomes were recorded (hospital admissions or ED attendance).

In order to identify predictors of the study outcomes univariate and multivariate regression analyses were conducted. The incidence of hospitalisations or ED attendance was analysed using mixed effects logistic regression, and the frequency of hospitalisations and ED attendances were analysed using negative binomial or Poisson regression, depending on the model fit based on overdispersion of the outcome, assessed separately for each model.

Initially, each potential predictor as detailed below was analysed separately. Each model additionally included an interaction term of the predictor with allocation to the E-SEE intervention or control group as a fixed effect to account for possible confounding by the intervention. Where such an interaction term resulted in non-convergence of the regression model, allocation was included as a main effect only. The infant’s age in weeks at baseline was also used as a fixed effect to account for the possible confounding by time involved in the study and therefore number of potential ED attendances or admissions. Recruitment site (1,2,3 or 4) was included as a random effect in the mixed effect logistic regression models and explored as a separate fixed effect in the Poisson and negative binomial models.

Statistically significant predictors (p < 0.05) from the univariate regressions were then included in a combined multivariate model for each outcome. Odds ratios (ORs) or Incidence Rate Ratios (IRRs) together with 95% confidence intervals (CIs) and p-values were reported for each predictor. All data were analysed on a complete case basis, with cases with any missing data for a given analysis excluded. The sample ranged from 324 to 341. All analyses were conducted using Stata 16 (Stata/SE 16.1 for Windows, StataCorp LLC).

## Results

A total of 341 mothers and 341 infants were included in the analysis. There were no twins in the study. Mothers’ average age was 31 years, were predominantly white British and in paid employment. Just over 40% were first time parents. The male/female split among infants was approximately even. They were recruited to the study aged between 2 and 8 weeks with a mean of 6 weeks. About 5% of babies had been born prematurely, and about a third had experienced health problems or serious injury since birth (Table 1).

**Table 1:** Mother and infant emergency department (ED) outcomes

	Total	Infant admitted to hospital at least once	Infant attended ED at least once	Mother admitted to hospital at least once	Mother attended ED at least once
	N = 341	N = 65	N = 145	N = 36	N = 43
**Mother**					
*Age (in years)*					
N	341 (100%)	65 (100%)	145 (100%)	36 (100%)	43 (100%)
Mean (SD)	30.9 (5.0)	30.0 (5.5)	30.3 (5.0)	28.8 (4.9)	30.5 (5.9)
Median (IQR)	31 (28, 34)	30 (26, 33)	30 (26, 33)	29 (25.5, 32)	30 (27, 35)
*Ethnicity*					
White/white British	274 (80%)	54 (84%)	126 (87%)	34 (94%)	32 (74%)
Asian/Asian British	50 (15%)	8 (12%)	15 (10%)	< 5	7 (16%)
Black/black British	8 (2%)	< 5	< 5	< 5	< 5
Mixed/multiple ethnic groups	8 (2%)	< 5	< 5	< 5	< 5
Other ethnic group	< 5	< 5	< 5	< 5	< 5
*Employment*					
Paid employed	250 (73%)	46 (71%)	104 (72%)	20 (56%)	25 (58%)
Unemployed	84 (25%)	17 (26%)	37 (26%)	14 (39%)	16 (37%)
Other	6 (2%)	< 5 (3%)	< 5 (3%)	< 5 (6%)	< 5 (5%)
Missing	< 5 (0.3%)	< 5 (0%)	< 5 (0%)	< 5 (0%)	< 5 (0%)
*Number of biological children (including current child)*					
1	147 (43%)	28 (43%)	72 (50%)	17 (47%)	20 (47%)
2	123 (36%)	23 (35%)	51 (35%)	10 (28%)	11 (26%)
3	44 (13%)	10 (15%)	15 (10%)	5 (14%)	8 (19%)
4–6	24 (7%)	< 5 (6%)	5 (4%)	< 5 (11%)	< 5 (9%)
Missing	< 5	< 5	< 5	< 5	< 5
*Age when first child was born (in years)*					
N	340 (99.7%)	65 (100%)	145 (100%)	36 (100%)	43 (100%)
Mean (SD)	27.5 (5.4)	26.3 (5.5)	27.6 (5.7)	25 (5.5)	26.9 (5.8)
Median (IQR)	28 (23, 31)	27 (23, 30)	28 (24, 32)	25 (21, 30)	27 (23, 31)
*History of emotional or physical abus*e					
Yes	40 (12%)	11 (17%)	22 (15%)	7 (19%)	8 (19%)
No	231 (68%)	38 (59%)	92 (64%)	15 (42%)	26 (61%)
Prefer not to answer	< 5 (1%)	< 5 (0%)	< 5 (0%)	< 5 (0%)	< 5 (0%)
Missing	67 (20%)	16 (25%)	31 (21%)	14 (39%)	9 (21%)
*EQ-5D-5L anxiety/depression*					
Not anxious or depressed	273 (80%)	46 (71%)	106 (73%)	23 (64%)	35 (81%)
Slightly anxious or depressed	51 (15%)	13 (20%)	29 (20%)	10 (28%)	5 (12%)
Moderately or severely anxious or depressed	17 (5%)	6 (9%)	10 (7%)	< 5	< 5
*PHQ-9 depression score*					
N	341 (100%)	65 (100%)	145 (100%)	36 (100%)	43 (100%)
Mean (SD)	3.0 (3.4)	3.7 (4.5)	3.2 (3.7)	4.9 (4.1)	3.7 (2.8)
Median (IQR)	2 (1, 4)	2 (1, 5)	2 (1, 5)	4 (2.5, 6)	3 (1, 6)
*PHQ-9 depression classification*					
No depression	241 (71%)	41 (63%)	98 (68%)	16 (44%)	25 (58%)
Mild depression	83 (24%)	18 (28%)	39 (27%)	16 (44%)	17 (40%)
Moderate or severe depression	17 (5%)	6 (9%)	8 (6%)	< 5	< 5
*First time parent*					
Yes	145 (43%)	28 (43%)	72 (50%)	16 (44%)	20 (47%)
No	196 (58%)	37 (57%)	73 (50%)	20 (56%)	23 (54%)
*Problems during pregnancy*					
Yes	148 (43%)	37 (57%)	71 (49%)	20 (56%)	26 (60%)
No	193 (57%)	28 (43%)	74 (51%)	16 (44%)	17 (40%)
*Problems/difficulties at the time of birth*					
Yes	183 (54%)	43 (66%)	83 (57%)	29 (81%)	26 (60%)
No	158 (46%)	22 (34%)	62 (43%)	7 (19%)	17 (40%)
**Infant**					
*Sex*					
Male	174 (51%)	33 (51%)	80 (55%)	22 (61%)	29 (67%)
Female	167 (49%)	32 (49%)	65 (45%)	14 (39%)	14 (33%)
*Gestational Age at baseline (in weeks)*					
N	338 (99%)	65 (100%)	145(100%)	36 (100%)	42 (98%)
Mean (SD)	39.6 (1.5)	38.8 (1.9)	39.3 (1.6)	39.5 (1.7)	39.5 (1.4)
Median (IQR)	39.7 (39.0,40.6)	39.0 (37.9,40.0)	39.3 (38.4,40.4)	40.0 (38.2,40.6)	40.0 (38.6,40.4)
*Born prematurely*					
Yes	12 (4%)	7 (11%)	8 (6%)	< 5	< 5
No	327 (96%)	57 (88%)	136 (94%)	35 (97%)	42 (98%)
Missing	< 5	< 5	< 5	< 5	< 5 (
*Health problems or serious injury since birth*					
Yes	103 (30%)	36 (55%)	51 (35%)	11 (31%)	10 (23%)
No	238 (70%)	29 (45%)	94 (65%)	25 (69%)	33 (77%)

There were 263 ED attendances (for 145 children, with up to 14 attendances per child) and 95 hospital admissions recorded (for 65 children, with up to 5 admissions per child) (Table 2). Admissions were related predominantly to respiratory conditions (40%) and other infectious diseases (21%).

**Table 2:** Summary of hospital admissions and ED attendances

	Mother		Infant	
	Hospital admissions	ED attendances	Hospital admissions	ED attendances
*Number of events/individuals*				
Number of events (N)	46	67	95	263
Number of individuals with one or more events (% total)	36 (10.6%)	43 (12.6%)	65 (19.1%)	145 (42.5%)
*Number of events per individual*				
N	36	43	65	145
Mean (SD)	1.3 (0.7)	1.6 (1.3)	1.5 (0.8)	1.8 (1.5)
Median (IQR)	1 (1, 1)	1 (1, 2)	1 (1, 2)	1 (1, 2)
Min, Max	1, 4	1, 8	1, 5	1, 14
*Length of stay (overnights)*				
N	36	–	65	–
Mean (SD)	6.2 (14.5)	–	3.2 (3.2)	–
Median (IQR)	2 (1, 5)	–	2 (1, 5)	–
Min., Max	1, 87	–	1, 19	–

Table 3 shows mother- and infant-related predictors of infant hospital admissions and ED attendances from the univariate and multivariate regression models.

**Table 3:** Predictors of infants’ emergency department admission and attendance

Outcome: At least one Hospital Admission (Infant)						
Predictor	Univariate			Multivariate		
	OR	95% CI	p-value	OR	95% CI	p-value
Problems during pregnancy (vs. no problems)	1.86	1.04 to 3.34	**0.037**	1.13	0.59 to 2.16	0.711
Child born premature (vs. born full term)	5.47	1.42 to 21.07	**0.014**	1.70	0.34 to 8.39	0.516
Child health problems or serious injuries since birth (vs no health problems)	3.70	2.01 to 6.81	** < 0.001**	3.25	1.76 to 6.01	** < 0.001**
Gestational age (in weeks)	0.72	0.60 to 0.88	**0.001**	0.78	0.61 to 1.00	0.050
Age of mother at birth of first child (in years)	0.94	0.89 to 0.99	**0.021**	0.97	0.92 to 1.03	0.376
Mother EQ-5D-5L score at baseline	0.02	0.00 to 0.53	**0.020**	0.14	0.00 to 4.73	0.270

Outcome: Frequency of hospital admissions (Infant)						
	IRR	95% CI	p-value	IRR	95% CI	p-value
Problems during pregnancy (vs. no problems)	1.80	1.04 to 3.11	**0.037**	1.21	0.68 to 2.15	0.510
Child born premature (vs born full term)	3.81	1.18 to 12.31	**0.026**	0.99	0.27 to 3.61	0.985
Difficulties at the time of child’s birth (vs. no problems)	2.00	1.12 to 3.56	**0.018**	1.71	0.97 to 3.01	0.062
Child health problems and serious injuries since birth (vs no health problems)	2.83	1.64 to 4.87	** < 0.001**	2.75	1.62 to 4.68	** < 0.001**
History of emotional or physical abuse by partner or someone important—over all follow-ups (vs. no history of abuse)	1.89	1.01 to 3.54	**0.048**	1.17	0.62 to 2.22	0.625
Gestational age	0.76	0.64 to 0.89	**0.001**	0.82	0.66 to 1.00	0.052
Age of mother (in years) at birth of first child	0.92	0.87 to 0.97	**0.001**	0.96	0.91 to 1.02	0.168

Outcome: At least one ED attendance (Infant)						
	OR	95% CI	p-value	OR	95% CI	p-value
First time parent (vs. existing parent)	1.80	1.11 to 2.93	**0.018**	1.06	0.49 to 2.28	0.878
Mother attending ED at least once during the study	2.10	1.00 to 4.39	**0.049**	2.34	1.13 to 4.84	**0.022**
Mother slightly, moderately or severely anxious or depressed based on baseline EQ-5D-5L sub-scale (vs. not depressed)	1.85	1.01 to 3.38	**0.046**	2.40	1.30 to 4.41	**0.005**
Age of mother at baseline (in years)	0.92	0.87 to 0.97	**0.001**	0.98	0.93 to 1.03	0.440
Gestational age (in weeks)	0.74	0.62 to 0.88	**0.001**	0.73	0.61 to 0.88	**0.001**
Number of biological children	0.72	0.56 to 0.94	**0.016**	0.69	0.45 to 1.06	0.090

Outcome: Frequency of ED attendances (Infant)						
	IRR	95% CI	p-value	IRR	95% CI	p-value
Site 1 (vs. Site 3)	0.61	0.37 to 0.99	**0.047**	0.46	0.29 to 0.73	**0.001**
Mother being unemployed (vs. paid employed)	1.52	1.01 to 2.30	**0.045**	1.15	0.75 to 1.77	0.517
First time parent (vs. existing parent)	1.59	1.10 to 2.29	**0.013**	1.25	1.88 to 1.79	0.213
Problems during pregnancy (vs. no problems)	1.45	1.01 to 2.29	**0.044**	1.31	0.92 to 1.88	0.134
Difficulties at the time of child’s birth (vs. no problems)	1.60	1.10 to 2.33	**0.013**	1.34	0.94 to 1.91	0.105
Child health problems and serious injuries since birth (vs. no health problems)	1.55	1.05 to 2.27	**0.026**	1.38	0.98 to 1.94	0.066
History of emotional or physical abuse by a partner or someone important—over all follow-ups (vs. no history of abuse)	1.55	1.02 to 2.34	**0.038**	1.12	0.75 to 1.66	0.582
Age of mother at baseline (in years)	0.95	0.91 to 0.98	**0.003**	0.96	0.92 to 1.00	**0.028**
Gestational age (in weeks)	0.85	0.76 to 0.96	**0.009**	0.90	0.80 to 1.01	0.074

Bold values are statistically significant (p < 0.05)

### Infants’ Emergency Department Attendance and Admissions

In univariate regressions significant predictors of hospital admission included physical problems during pregnancy, the child being born prematurely and child health problems since birth. In a combined regression using all significant univariate predictors, only ‘Child health problems or serious injury since birth’ remained statistically significant (OR 3.25, 95% CI 1.76 to 6.01, p < 0.001). A similar pattern was observed for the frequency of hospital admissions, with only child health problems and serious injury remaining statistically significant in the combined regression model (IRR 2.75, 95% CI 1.62 to 4.68, p < 0.001).

In the combined regression model ED attendances were more likely among infants with lower gestational age (OR 0.73, 95% CI 0.61 to 0.88, p = 0.001), whose mothers had anxiety or depression on the EQ-5D-5L (OR 2.40, 95% CI 1.30 to 4.41, p = 0.005) and whose mother had attended the ED at least once during the trial (OR 2.34, 95% CI 1.13 to 4.84, p = 0.22). The frequency of infant ED attendance was associated with the age of the mother (IRR 0.96, 95% CI 0.92 to 1.00, p = 0.028) and geographical site (IRR 0.46, 95% CI 0.29 to 0.73, p = 0.001; summary effect: χ^2^ (df = 3) = 10.02, p = 0.018) in the multivariate regression.

### Mothers’ Emergency Department Attendance and Admissions

For the mothers, there were a total of 67 ED attendances (for 43 mothers, up to 8 per person) and 46 emergency hospital admissions recorded (for 36 mothers, up to 4 per person) (Table 2). Admissions were related predominantly to reproductive health (61%), surgery (13%) and other infectious diseases (11%). Table 4 illustrates predictors of mothers’ hospital admissions and ED attendances from the univariate and multivariate regression models.

**Table 4:** Predictors of mothers’ emergency department admission and attendance

Outcome: At least one Hospital Admission (Mother)						
	Univariate			Multivariate		
	OR	95% CI	p-value	OR	95% CI	p-value
Unemployed (vs paid employed)	2.40	1.09 to 5.28	**0.030**	1.08	0.37 to 3.17	0.885
Problems or difficulties at the time of child's birth (vs. no problems or difficulties)	4.07	1.73 to 9.59	**0.001**	2.18	0.82 to 5.83	0.119
Mother having attended the ED department at least once during the study (vs having not attended)	4.15	1.76 to 9.74	**0.001**	2.36	0.84 to 6.62	0.104
Mother slightly, moderately or severely anxious or depressed based on baseline EQ-5D-5L sub-scale (vs not anxious or depressed)	3.34	1.53 to 7.28	**0.002**	2.31	0.80 to 6.65	0.120
History of emotional or physical abuse by a partner or someone important—Over all follow-ups (vs. no history of abuse)	2.78	1.27 to 6.09	**0.011**	1.92	0.72 to 5.12	0.190
Age of mother at baseline (in years)	0.91	0.85 to 0.98	**0.017**	0.96	0.87 to 1.06	0.415
Age of mother when first child was born (in years)	0.93	0.86 to 0.99	**0.027**	0.99	0.88 to 1.10	0.803
PHQ-9 score at baseline	1.13	1.04 to 1.23	**0.004**	1.08	0.94 to 1.23	0.273

Outcome: Frequency of hospital admissions (Mother)						
	IRR	95% CI	p-value	IRR	95% CI	p-value
Unemployed (vs. paid employed)	2.89	1.34 to 6.24	**0.007**	1.16	0.53 to 2.52	0.715
Problems or difficulties at the time of child's birth (vs. no problems or difficulties)	3.45	1.47 to 8.08	**0.004**	2.02	0.88 to 4.61	0.097
Mother having attended the ED department at least once during the study (vs. having not attended)	4.29	1.90 to 9.69	** < 0.001**	3.39	1.66 to 6.93	**0.001**
Mother slightly, moderately or severely anxious or depressed based on baseline EQ-5D-5L sub-scale (vs. not anxious or depressed)	3.70	1.74 to 7.85	**0.001**	3.10	1.14 to 8.45	**0.027**
History of emotional or physical abuse by a partner or someone important—Over all follow-ups (vs. no history of abuse)	2.57	1.17 to 5.62	**0.019**	1.51	0.73 to 3.14	0.268
Age of mother at baseline (in years)	0.89	0.83 to 0.95	**0.001**	0.98	0.91 to 1.05	0.516
Age of mother when first child was born (in years)	0.91	0.85 to 0.97	**0.007**	0.97	0.90 to 1.06	0.547
PHQ-9 score at baseline	1.15	1.05 to 1.27	**0.003**	1.08	0.99 to 1.18	0.072

Outcome: At least one ED attendance (Mother)						
	OR	95% CI	p-value	OR	95% CI	p-value
Mixed or multiple ethnic group (vs. white British ethnicity)	7.47	1.78 to 31.42	**0.006**	9.62	2.19 to 42.27	**0.003**
Having a male child (vs. female child)	2.34	1.10 to 4.99	**0.028**	2.08	1.03 to 4.20	**0.042**
Mother being hospitalised at least once during the study (vs. not being hospitalised)	4.15	1.76 to 9.78	**0.001**	4.15	1.81 to 9.56	**0.001**

Outcome: Frequency of ED attendances (Mother)						
	IRR	95% CI	p-value	IRR	95% CI	p-value
EQ5D-5L score (baseline)	0.01	0.00 to 0.72	**0.048**	–	–	**–**

Bold values are statistically significant (p < 0.05)

In univariate regressions, hospital admission was related to age at baseline (OR 0.91, 95% CI 0.85 to 0.98, p = 0.017), being unemployed (OR 2.40, 95% CI 1.09 to 5.28, p = 0.030), had attended ED at least once during the study (OR 4.15, 95% CI 1.76 to 9.74, p = 0.001), had a history of emotional or physical abuse (OR 2.78, 95% CI 1.27 to 6.09, p = 0.011), were more anxious or depressed based on the EQ-5D-5L (OR 3.34, 95% CI 1.53 to 7.28, p = 0.002) and PHQ-9 (OR 1.13, 95% CI 1.04 to 1.23, p = 0.004), and had problems during delivery (OR 4.07, 95% CI 1.73 to 9.59, p = 0.001). However, in the combined multivariate model none of the predictors remained statistically significant. Of note is that the most missing data (n = 17) was for questions related to intimate partner violence.

The frequency of hospital admission for mothers was associated with similar factors in the univariate model; having attended ED at least once during the trial (IRR 3.39, 95% CI 1.66 to 6.93, p = 0.001), and being slightly, moderately or severely depressed based on the EQ-5D-5L (IRR 3.10, 95% CI 1.44 to 8.45, p = 0.027) remained statistically significant in the multivariate model.

Significant predictors of ED attendance were the same in the univariate and multivariate models. In the multivariate model ED attendance was more likely for mothers of mixed or multiple ethnicity (OR 9.62, 95% CI 2.19 to 42.27, p = 0.003), if the index child was a boy (OR 2.08, 95% CI 1.03 to 4.20, p = 0.042), and for those who had been hospitalised at least once during the study (OR 4.15, 95% CI 1.81 to 9.56, p = 0.001). Regarding the frequency of attendance at the ED, only having a higher EQ-5D-5L score (IRR 0.01, 95% CI 0.00 to 0.72, p = 0.048) was statistically significant in the univariate model.

## Discussion

This study explored the predictors for emergency department (ED) attendance and hospital admission for mothers and their infants enrolled in a study to improve social and emotional health. We found that ED attendance of the infant was predicted by their younger gestational age and mothers’ poorer mental health and their mothers having attended to the ED at least once during the study. Frequency of ED attendance was predicted by site, and age of their mother. Health problems or injury since birth predicted their hospital admission. They were most often admitted for respiratory or other infectious disease.

For mothers, ED attendance was predicted by mixed ethnic origin, having a boy child, and having been hospitalised during the study. They were most commonly admitted to hospital for reproductive health issues and admission to hospital was predicted by previous attendance at the ED and anxiety or depression.

The study benefits from a comprehensive data set with few missing data. However, the limited sample size meant that we had reduced power in the multivariate analyses. The report of relationship abuse was likely to be underreported.

A previous study of a similar size found no link with maternal mental health and infant health care utilisation although it excluded women with high risk pregnancies (Anderson et al., 2008). Our analyses provide more detail than previous studies. Psychosocial stress is known to be associated with obstetric complications, including pre-term birth (Loomans et al., 2013) and depressive symptoms which have been reported to be related to other adverse pregnancy and post-pregnancy outcomes (Alvarez et al., 2015). Additionally, poor preconceptual mental health is also known to be related to adverse obstetric outcomes (Witt et al., 2012). Both maternal and paternal mental health are associated with an increase in child mortality and suboptimal cognitive and developmental trajectories during both childhood and adolescence (Walker et al., 2011).

Previous studies have suggested that maternal health care utilisation is associated with infant health care utilisation. Mothers’ use of health care services is influenced by their beliefs about the service and can influence their decisions to use these services for their infant/s. However, as the child gets older the child’s own health status plays a more important role (Newacheck & Halfon, 1986). A relationship between *parental* and childhood ED utilisation has also been shown previously (Mistry et al., 2005).

Previous research has also suggested that poor continuity of primary care is associated with higher risk of ED utilisation and hospitalisation (Christakis et al., 2001). The connection between ED hospital use by mothers and infants is an important finding and suggests that we consider the importance of integrated care services for families as a whole. Maternal mental and physical health problems would normally lead to primary care intervention in the NHS in England. However, recent reports have suggested that the universal health service has been compromised and general and nurse practitioner services have become less accessible for some families (Whittaker et al., 2021).

Minoritised ethnicity has been shown to play a part in access to emergency care services, though while other studies have shown a trend towards less likely use of emergency services (Hull et al., 1997) we found it predicted ED use. Less accessible primary and community care services may contribute to an increase in the use of EDs for health care. Clearly this relationship is complex and requires more nuanced in-depth qualitative methodologies.

The main diagnoses for infants’ admission to hospital are similar to the most common conditions registered in England between 2015 to 2016 (Keeble & Kossarova, 2017). However, this paper additionally contributes to the literature on preterm infants and emergency hospitalisation, (Kuzniewicz et al., 2013) demonstrating a link with gestational age of the infant. Early gestational age has also been linked to respiratory disorders (Laughon et al., 2011).

Although these data were collected before the COVID-19 pandemic, recent evidence suggests that emergency attendance for mothers during pregnancy, (Anteby et al., 2021) and attendance of their infants (Dann et al., 2020) has been delayed and that primary health care provision and access has been further reduced, (Saunders & Hogg, 2020) which could lead to poorer maternal and child health outcomes in the coming years.

A complex interplay of maternal and infant health factors predicts ED attendance and admission. Continuous integrated health care services during pregnancy and beyond can have a positive impact (Baxter et al., 2018; Kerber et al., 2007). Therefore, we should continue to support high quality, accessible, preventative services throughout pregnancy to ensure that mental and physical health needs are addressed as soon as they arise.

## Data Availability

Anonymised data from this study are available from the corresponding author. If you are interested in accessing these data, a proforma outlining your proposal will be required. Your request will be reviewed by the study team. We will endeavour to respond to requests as soon as possible. The lead author (the manuscript's guarantor) confirms that the manuscript is an honest, accurate, and transparent account of the study being reported, that no important aspects of the study have been omitted and any discrepancies from the study as originally planned have been explained.
